# Effectiveness of Common Extraction Solvents in Obtaining Antioxidant Compounds from African Medicinal Plants

**DOI:** 10.3390/antiox14121498

**Published:** 2025-12-13

**Authors:** Khayelihle Ncama, Joseph Malele, Dhiren Munsami Govender, Thagen Anumanthoo, Mack Moyo

**Affiliations:** Department of Horticulture, Durban University of Technology, Durban 4000, South Africa

**Keywords:** phytoextracts, antioxidants, African herbs, sustainability, medicinal plants

## Abstract

The efficacy of phytoextracts is equally affected by the extraction solvent and the extraction method. Details of the solvent type, concentration, density, and other characteristics are associated with the quality of the resultant extract. Some solvents have been found to be effective only on specific parts of plants. Industry has shown a growing interest in eco-friendly plant extracts for the formulation of medication, food additives, cosmetics, and agricultural products. This interest is aligned with the proven necessity of sustainability, marketability, and regulation of manufactured products in value chains. In this review, the literature on antioxidant compounds and activity of extracts from African medicinal plants is reviewed. Findings indicate that the use of ethanol, methanol, water, and to a lesser extent, acetone as solvents for the extraction of antioxidant compounds is common. The use of these solvents is supported by decisive selection of procedure, ideal temperature, duration, solvent pH, and the extracted plant parts. Fermentation enhances the antioxidant activity of aqueous extracts but reduces that of alcohol extracts. This is particularly essential in continents such as Africa, where water is available but alcohol is scarce. “Green” extraction technologies are not as successful as solvent extractions for use with African medicinal plants. There is a financial hurdle that results in a mismatch between academic research innovations and societal transmission to new technologies, as most communities are dominated by small-to-medium enterprises. Further studies on the extraction of antioxidants from African medicinal plants are recommended to guide the research and link it to ordinary African societies.

## 1. Introduction

The use of medicinal herbs to boost the human immune system or remedy ailments is a well-received concept compared to the use of Western remedies in Africa [1]. The culture of using plant-derived immune boosters dates back centuries before colonialism [2]. Many people in rural areas use aqueous plant extracts for various medicinal benefits including immune boosters, curing diseases, steaming for skin purification, and cleaning the digestive system. The preferred use of water during extraction of antioxidant compounds from African medicinal plants has limitations such as low solubility, cyto-percolation strength, and impurity of extracts. These limitations of using water have led to experiments mixing it with various solvents including alcohols and acids [3]. Research has shown that cellular imbalance between antioxidant compounds and free radicals from strong inorganic solvents can lead to oxidative stress that results in various health issues including accelerated aging, cancer, and heart disease [4]. The United Nations’ primary sustainable development goals have made natural extraction techniques increasingly important. Unconventional extraction techniques and natural solvents are being used to create greener extraction procedures that are less toxic, leading to less pollution of the environment [5]. Various techniques for extracting antioxidant compounds from African medicinal plants have been reported in the literature, including the use of solvents in combination with the latest technologies that leave a low ecological footprint [6]. Recently discovered technologies such as microwave-assisted, supercritical fluid, ultrasonic-assisted, and pressurized liquid extractions are aligned with improved efficiencies and rapidness of the extraction process [7].

However, Africa is dominated by developing countries that cannot afford advanced technologies, which results in the use of solvent extraction for obtaining different antioxidant compounds from medicinal plants. For example, the extraction of total phenolic and flavonoid compounds from Sudanese medicinal plants, namely *Blepharis linariifolia*, *Cyperus rotundus*, *Guiera senegalensis*, *Maerua pseudopetalosa*, *Tinospora bakis*, and *Dicoma tomentosa* was carried out using solvents of different polarity: 50% ethanol (EtOH), 70% EtOH, 95% EtOH, acetone, and dichloromethane [8]. Chloroform, ethyl acetate, and methanolic and aqueous extracts were used during the extraction of phytochemical and antioxidant compounds of medicinal plants (*Azadirachta indica* A. Juss and *Vernonia amygdalina* Del) in Malawi [9]. The phytochemical and antioxidant profiling as well as the antibacterial activities of *Cordia africana* were reported from its methanolic extracts in Ethiopia [10].

The profiling of antioxidant compounds in medicinal plants can be associated with their phytoremediation or antimicrobial capacities. As such, the assessment of antimicrobial activity of extracts has been widely used concurrently with antioxidant compounds to demonstrate the usefulness of plant extracts in research into medicinal plants. Dichloromethane and methanolic extracts from 101 medicinal plants in South Africa were previously demonstrated to contain antimutagenic properties associated with the plants’ antioxidant activity, especially from methanolic extracts, which were superior to dichloromethane extracts [11]. Mabona et al. [12] found that dichloromethane and methanolic extracts from 47 different South African medicinal plantsfrom 38 families exerted antimicrobial activity and had ethnopharmacological properties, which were correlated to the obtained concentrations of bioactive compounds. Numerous studies have been executed on the phytomedicinal efficacy of medicinal plant extracts. Methanolic extracts from 29 medicinal plants of northern Angola were demonstrated to have anti-inflammatory properties [13]. In Cameroon, crude aqueous extracts from *Discoglypremna caloneura*, *Trichilia dregeana*, *Detarium microcarpum*, *Persicaria capitata*, and *Tapinanthus bangwensis* were found to inhibit hepatitis C virus in vitro [14]. Dichloromethane and methanolic extracts from 17 plants including *Daniellia oliveri*, *Eleusine africana*, *Lippia dulcis*, *Gardenia ternifolia*, *Senecio madagascariensis*, *Sterculia tragacantha*, *Tetracera alnifolia*, *Pontederia crassipes*, *Gardenia ternifolia*, and *Tillandsia albida* exhibited antimicrobial activities in Guinea [13]. In addition to the association of total antioxidant compounds and antimicrobial activities, research on the link between antimicrobial activity and concentrations of specific antioxidant compounds has been carried out. Positive correlations between phenol content and antibacterial activity of *Daphne gnidium* L., *Ajuga iva* L. Schreb, *Lavandula stoechas* L., *Cistus albidus* L., and *Cistus monspeliensis* L. ethanolic extracts were reported in Morocco [15]. Thus, the use of extraction solvents is a cross-continental practice that has been used to demonstrate both the antioxidant compounds and the antioxidant capacity of various medicinal plants. The literature shows the popularity of ethanol, methanol, and water as the most common solvents for extraction of antioxidant compounds from African medicinal plants [14,15,16].

Most authors have highlighted the correlation of antioxidant compounds’ concentration with the extraction technique or the type of solvent that is used. However, there is a need for increased awareness and comparison of extraction procedures to guide sustainability, affordability, effectiveness, and acceptability, especially in the communities of Africa. Researchers into the extraction of phytochemicals from medicinal plants have raised common suggestions for extraction of biologically active plant compounds via “green” technologies, and this has become a global aim in studies of phytochemical compounds [17]. However, there is a need for regional reports that highlight the status and the rate of progression towards green technology, particularly in the developing countries of Africa, to facilitate development. In this review, the research into antioxidant compounds and the activities of extracts from African medicinal plants is reported by reviewing the recent literature. This paper reports popular solvents that result in effective extracts with antioxidant capacity that may be associated with antimicrobial activity. This knowledge can guide acceptance and overcome hurdles in the adoption of recent innovative “green” technologies.

## 2. Ethanolic Extraction

Plants are a known source of antioxidants such as phenolic compounds (flavonoids, tannins, stilbenes, lignans, coumarins and phenolic acids), vitamins (A, C, and E) as well as carotenoids [18]. Obtaining these compounds requires a well-executed extraction process, including the selection of a solvent. Ethanol is amongst the popular solvents used in the extraction of natural substances, and it is reported to produce extracts that are safe for human consumption [19]. Anokwuru et al. [20] evaluated the effect of extraction solvents including methanol, ethanol, ethyl acetate, and acetone on phenolic compounds, flavonoid concentrations, and antioxidant activity from *Azadirachta indica* bark, *Acalypha wilkesiana* leaves, and *Solanum scabrum* leaves. The authors found that the absolute ethanol extract from *A. indica* exhibited the highest flavonoid content (8.7 g quercetin equivalent (QE)/100 g) compared to ethyl–acetate extract (5.32 g QE/100 g), acetone extract (5.15 g QE/100 g), and methanol extract (5.14 g QE/100 g). Flavonoids have been reported to scavenge reactive oxygen species due to the presence of their phenolic hydroxyl groups that make them strong antioxidant compounds [20].

Dirar et al. [8] investigated the effect of extraction solvents on the total phenolic and flavonoid contents as well as biological activities of extracts from Sudanese medicinal plants. It was found that the extracts acquired using 50% ethanol, 70% ethanol, and 70% acetone had the highest total phenolic content [8]. With regards to antioxidant activity, most of the 70% and 50% ethanol extracts displayed higher free radical scavenging activity compared to water and dichloromethane extracts. In the DPPH radical scavenging assay, the 50% ethanol extract of *Guiera senegalensis* showed the highest activity (IC50 = 11.36 ± 1.08 μg/mL), which was comparable to the positive control (IC50 = 10.12 ± 0.03 μg/mL). The study attributed the antioxidant activity of 70% and 50% ethanol extracts to the higher concentrations of phenolic and flavonoid compounds obtained. Mansour et al. [21] reported a high concentration of phenolic compounds using ethanol as a solvent. The antioxidant activity of *Ormenis africana* hydroethanolic extract (20:80 ratio) displayed high polyphenol content (312.07 mg gallic acid equivalent (GAE)/g DM), anthocyanin content (0.28 ± 0.09 mg cynadin-3-glucoside (C3G)/g DM), and flavonoid content (73.72 ± 1.98 mg QE/g DM), indicating that the extract had considerable levels of antioxidant compounds [22]. Furthermore, higher antioxidant activity was found in the 2,2-diphenyl-1-picrylhydrazyl (DPPH) than the ABTS (2,2′-azino-bis (3-ethylbenzothiazoline-6-sulfonic acid)) assay (IC50 = 24 μg/mL; TEAC = 2.137 mM), indicating the potential of *Ormenis africana* as a source of antioxidants.

Adebiyi et al. [22] used different in vitro models to evaluate the free radical scavenging activity of ethanolic extracts from stems and leaves of *Grewia carpinifolia*. The extracts effectively scavenged ABTS radicals (IC50 = 0.32 mg/mL for leaf extract and 1.98 mg/mL for stem extract), and it was also found that the percentage of inhibition increased with increasing extract concentration. This trend was also noted in the DPPH assay, where there was an increase in scavenging activity of DPPH radicals (IC50 = 0.39 mg/mL for leaf extract and 0.81 mg/mL for stem extract), as the concentration of the plant extracts increased. Notably, the scavenging of ABTS radicals was higher in comparison to the scavenging of DPPH radicals by the extracts. Both the leaf and stem extracts displayed significant antioxidant activity in all conducted assays [22]. Specifically, the stem extract exhibited higher antioxidant activity in the ABTS assay whilst the leaf extract displayed a higher antioxidant activity across all other antioxidant assays. The study also found that ethanolic extracts of *Grewia carpinifolia* included a substantial amount of phenolic compounds which the study deemed responsible for the antioxidant activity. It is evident from the findings that the selection of an appropriate extract assessment method is more important than choosing a plant part to be extracted. Ethanolic extracts from various modifications have been reported to result in high extraction of antioxidant compounds (Table 1).

### Duration of Ethanolic Extraction

Several factors influence the extraction of phenolic compounds from plants. However, the duration of extraction is considered amongst the most important [23]. Conventional extraction methods (Soxhlet extraction, maceration, and heat-assisted extraction) are associated with longer extraction times and higher solvent usage while advanced extraction methods involving high pressure, ultrasound, enzymatic hydrolysis, high voltage, microwaves, adsorption, mechanical forces, and innovative solvents are associated with shorter processing times and lower solvent usage. Advanced extraction methods are also associated with higher yields, better energy efficiency, and higher selectivity, and have the potential to avoid organic solvents [24]. A study by Dzieciol [25] evaluated the effect of extraction techniques on the yield and antioxidant activity of ethanolic *Moringa oleifera* leaf extracts. The study compared three extraction techniques (maceration with shaking, ultrasound-assisted extraction (UAE), and Soxhlet extraction (SE)) conducted across different time variants (1, 2, and 4 h). The highest extraction yield of antioxidant compounds was obtained using UAE and SE with a 4 h extraction time, whereas the least effective duration was the 1 h SE [25]. However, the highest phenolic content and antioxidant activity was obtained in the 1 h UAE. There was a decrease in the total phenolic content and antioxidant activity observed in the UAE and SE systems when extraction time was increased, which was associated with biologically active compounds’ possible decomposition due to the influence of ultrasound or increased temperatures.

Chew et al. [26] demonstrated the effect of ethanol concentration, extraction time, and temperature on the recovery of phenolic compounds and the antioxidant capacity of *Orthosiphon stamineus* extracts. The study found that the total phenolic content and the content of condensed tannins decreased when the extraction time was longer than 240 and 120 min, respectively. Chew et al. [26] attributed these findings to the oxidation of phenolic compounds due to prolonged exposure to light or oxygen resulting in a decrease in phenolic content in the crude extract. It was further stated that prolonged extraction times are not useful for extracting phenolic compounds from *O. stamineus*. This relates to Fick’s second law of diffusion which explains that as the extraction process progresses, equilibrium will be achieved between the extraction solvent and the solute in the plant sample [27].

This information is important in the determination of both antioxidant compounds and resultant medicinal activity. Wendakoon et al. [28] found that the antibacterial activity was dependent on different concentrations of ethanol on hops, buchu, oregano, and grape. About 90% of *Humulus lupulus* ethanolic extracts were found to be active against *Staphylococcus aureus*, while 50% buchu leaf performed better than higher levels of ethanol extraction. A direct proportional relationship is noted between the concentration of the extract and antioxidant activity. Ethanolic extracts from plants can have different capacities to react and reduce various radicals. Notably, more research can be conducted into the duration of ethanolic extraction and obtainable antioxidant activities from African medicinal plants.

**Table 1:** Extraction of antioxidant compounds from African medicinal plants using ethanolic solvents.

Plant Species	Solvent/Method	Key Findings	Citation
*Azadirachta indica* (bark), *Acalypha wilkesiana* (leaves), *Solanum scabrum* (leaves)	Ethanol	The ethanol extract of *Azadirachta indica* showed the highest flavonoid content.	[20]
*Guiera senegalensis* and other Sudanese medicinal plants	50% ethanol, 70% ethanol, acetone	50% and 70% ethanol extracts had the highest total phenolic content and displayed higher free radical scavenging activity. The 50% ethanol extract of *Guiera senegalensis* showed the highest DPPH radical scavenging activity.	[8]
*Ormenis africana*	Hydroethanolic extract	Displayed high polyphenol, anthocyanin, and flavonoid content, indicating significant antioxidant activity.	[21]
*Grewia carpinifolia* (stems and leaves)	Ethanolic extracts	The extracts effectively scavenged ABTS and DPPH radicals in a concentration-dependent manner. The stem extract showed higher activity in the ABTS assay.	[22]
*Moringa oleifera* (leaves)	Ethanolic extracts (maceration, ultrasound assisted extraction, Soxhlet)	The highest phenolic content and antioxidant activity were obtained with a 1 h ultrasound-assisted extraction (UAE). Longer extraction times led to a decrease in active compounds associated with alcohol evaporation or reaction with the active compounds.	[25]
*Orthosiphon stamineus*	Ethanolic extracts	Prolonged extraction times (over 240 min for phenolics, 120 min for tannins) led to a decrease in compound recovery due to oxidation.	[26]
*Typha capensis*	Water, methanol, ethanol, acetone, hexane	Ethanol crude extract yielded the highest phenolic content, while the acetone extract showed good antioxidant activity against DPPH and ABTS.	[29]

## 3. Methanolic Extraction

The solvent used during extraction is known to have a significant effect on the nature and type of secondary metabolites extracted from medicinal plants [8]. Natural phenols play a significant role in treating human health diseases, as evident from their antifungal, antioxidant, and anti-cancer activities [11]. According to Banso and Adeyemo [30], compounds derived from plants such as tannins are responsible for antioxidant and antimicrobial activity against bacteria and fungi. Moreover, the extraction method or technique used plays an important role in the types of phytochemicals produced [8,31]. The effectiveness of the procedure depends on the plant type, the plant part used, solvent type, and its concentration [32]. For instance, methanolic extraction of *Amaranthus hybridus* Linn leaves had a total phenolic content of 40.1 mg GAE/g DM, which was higher than the seed, which had total phenolic content of 31.20 mg GAE/g DM. The total phenolic content of *Anacardium occidentale* was found to be high when methanol extract of the stem bark was used instead of water or ethanol [33]. In *Calpurnia aurea*, the methanol extracts of stem and leaves revealed that the antioxidant activity of the stem extracts determined by the total phenol, flavonoids, and FRAP methods was higher than that of the leaves [34]. The dose response curve indicated that the leaf extracts exhibited higher DPPH radical scavenging activity compared to the stem extracts. Methanolic leaf and stem extracts of *Calpurnia aurea* were found to be fast and effective scavengers of ABTS radicals, and the activity was comparable to that of BHT [34]. The methanolic extraction of stem and leaves of *Celtis africana* determined by the DPPH revealed higher proanthocyanidins, total phenols, flavonoids, and total flavonols on the stem compared to the leaves, while the FRAP content were higher on the leaves than that of the stem [35]. When comparing acetone, methanol, and water with regard to the antioxidant activity of *Leonotis leonorus* and *Solanum nigrum*, the acetone extracts from *S. nigrum* had higher levels of polyphenols than the other extracts [11]. However, methanol extracts had higher proanthocyanidin content than the other solvent extracts.

The ferrous reducing antioxidant power (FRAP) values of the acetone and methanol extracts of *S. nigrum* were higher than those of BHT [11]. The FRAP values of the three extracts of *L. leonorus* were higher than those of BHT but lower than those of catechin (972 µmol Fe (II)/g), ascorbic acid (1632.1 µmol Fe (II)/g), and quercetin (3107.3 µmol Fe (II)/g). The acetone crude extract of *Typha capensis* was found to have good antioxidant activity against DPPH and ABTS, with IC50 values of 7.11 ± 1.7 µg/mL and 2.49 ± 2.02 µg/mL, respectively. However, the highest extraction yield was obtained with water, followed by methanol, ethanol, acetone, and hexane, respectively, but the highest phenolic content (45.29 ± 0.86 mg GAE/g) was attained with ethanol crude extract [30].

The antioxidant capacity of methanolic extracts from *Tetrapleura tetraptera* and *Parkia biglobosa* species was found to exhibit higher values compared to ethanolic and water extracts [30]. The antioxidant capacity correlated with polyphenol content, with *Tetrapleura tetraptera* exhibiting higher antioxidant capacity than *Parkia biglobosa*. However, for both species, the ethanol and water extracts at 1% exhibited higher values in all concentrations examined when assessing the antioxidant activity using DPPH. The highest reduction capabilities for both fruit species were observed at 0.3 mg/mL, with methanol extract of *Tetrapleura tetraptera* exhibiting the highest reduction potential. In all cases, the absorbance values correlated with the polyphenol content and antioxidant capacities [30]. *T. tetraptera* and *P. biglobosa* are widely used in West Africa for their nutritional and medicinal value. In a study by Konan et al. [36], the highest phenolic compound yield of 30.8% was obtained from the methanol extraction of *Psorospermum febrifugum* and the lowest yield of 1.3% was obtained from dichloromethane extract. The phenolic compound yield was high on *Myrianthus arboreus* methanolic extract yielding 8.4% and 0.8% from the dichloromethane extract. The antioxidant quality was determined by high radical scavenging activity of the methanolic extract of *P. febrifugum* (IC50 = 2.3 µg/mL), which exhibited a value lower than ascorbic acid (IC50 = 2.9 µg/mL) and higher total phenolic compounds [36]. Unfermented methanolic *Aspalanthus linearis* extracts showed higher concentrations of phenolic compounds, and the mean for fermented was 16% lower (257 for fermented vs. 303 GAE/g for unfermented), while water extracts had similar phenolic distributions with nearly identical means (279 for fermented vs. 282 GAE/g for unfermented) [37]. However, the fermented water extracts had higher content compared to fermented methanolic extracts. This finding highlights the importance of using processed water as a solvent rather than using pure water. It is possible that some water isotopes can aid in cellular penetration and cause cell-wall disruptions, leading to higher concentrations obtained from extracts.

Methanolic extracts of *Achillea millofelium*, *Bergenia ciliata*, and *Aloe vera* exhibited moderate DPPH/ABTS radical scavenging activity with IC50 values of 72.33 ± 0.73, 60.27 ± 0.20, and 77.89 ± 0.67 µg/mL, respectively, compared to the standard L-ascorbic acid (35.66 ± 0.56 µg/mL). The highest antioxidant activity from samples extracted with methanol solvent showed a relationship with more polar constituents such as bergenin, gallic acid, tannin acid, catechin, 3-O-galoylcatechin, and -3-O-galloylepicatechin found in the rhizome of *B. ciliata* [38]. The type of chemical constituents obtained from these extracts depends on the nature of the plant material, its source, the extent of processing, and the moisture level, as well as the particle size of the ground plant material. Moreover, factors that affect the secondary metabolite composition and quality in the extract include type and time of extraction, the nature and concentration of the solvent, processing temperature, and the polarity of the analytes [32]. Solvent extracting power, extraction duration and, to a lesser extent, their interaction, exerted a significant effect on the total polyphenol levels and antioxidant activity of *Mesembryanthemum edule* shoots [39]. In the study, methanolic extract was richer in total polyphenols (105 mg GAE/g DW) than ethanolic extract (75 mg GAE/g DW). Increasing the duration of sonication from 5 to 10 min enhanced the polyphenol contents of ethanol and methanol extracts by 32 and 81%, respectively. The methanolic extract obtained by longer duration of 10 min produced greater scavenging activity (IC50 750 µg/mL) compared to 5 min (IC50 788 µg/mL), as well as the iron reducing capacity, with IC50 of 234 and 255 µg/mL, respectively [40].

## 4. Methanolic Extracts and Bioactivity

Medicinal plants have been used traditionally as antibacterial agents and are well accepted today as a source of antioxidants [41]. Methanolic extracts from the leaves of *Napoleona imperialis*, *Psidium guadjava* and the roots of *Anthocleista djalonensis* demonstrated remarkable growth inhibitory activity against multi-resistant Gram-positive and Gram-negative wound isolates (*Staphylococcus aureus* (four strains), *E. coli* (two strains), *Pseudomonas aeruginosa* (one strain), *Proteus* spp. (three strains), and *Shigella* spp. (one strain) and demonstrated wound-healing properties comparable to the antibiotic powder Cicatrin [42]. These plants were previously reported to have various healing properties for several ailments including cough, malaria, and wounds [42]. The healing properties of the plants can be aligned with their high antioxidant properties [43]. In terms of bioactivity, the use of methanol in the leaf extraction of *Rhoicissus digitata* and root extraction of *Rhoicissus rhomboideae* was found to be effective and consistently exhibited the highest activity against *Candida albicans* [44]. Plants produce secondary metabolites such as phenolic compounds when they are stressed, and these phenolic compounds act as inhibitors of fungal enzymes and oxidative processes [45]. The methanolic extraction of five medicinal plants studied in south-west Morocco (*Pistacia atlantica*, *Cistus villosus*, *Ononis natrix*, *Rosa canina*, and *Lawsonia inermis*) were found to have significant antifungal activity due to a high content of polyphenols and a strong antioxidant effect [46].

Methanolic extracts of Vitaceae family plants such as *Cyphostemma natalitium* root, *Rhoicissus digitata* leaf, and *Rhoicissus rhomboidea* root showed significant inhibitory activity against COX-1 compared to 89% inhibition by the indomethacin standard [44]. The use of polar solvents appears to be effective in extracting compounds containing antioxidant activity, as observed in the methanolic extraction of *Zanthoxylum davyi* roots which showed good acetylcholinestarase (AChE) with the lowest IC50 value of 0.01 mg/mL [47]. Apart from *Terminalia sericea*, the ethyl–acetate extracts of all the assessed plants showed no activity or low radical scavenging activity in both the DPPH and ABTS assays, as indicated by their IC50 values, whilst the methanolic extracts showed higher antioxidant activities [47]. A 50% methanolic extraction of South African medicinal plants such as *Tedradenia riparia* and *Trichilia dregeana* that had been stored for 12 to 16 years exhibited significantly greater acetylcholinesterase inhibition compared to freshly harvested materials [48]. Out of six species investigated for antifungal activity of methanolic extracts, four plants (*Bucida buceras*, *Hapephyllum caffrum*, *Vanguaria infausta*, and *Xylotheca kraussiana*) had good antifungal activity against three sensitive fungi, with minimum inhibitory concentration (MIC) values as low as 0.2 mg/mL and 0.08 mg/mL against *Penicillium janthinellum*, *Trichoderma harzianum*, and *Fuzarium oxysporum* [49]. Plant extracts with low MIC values could be regarded as good sources of bioactive components with antimicrobial activity. That study found that acetone was the most effective solvent compared to methanol, but methanolic extracts are commonly used in antifungal studies. Satisfactory antifungal activity was observed with methanol extracts of *Rubus rigidus* (5 mm inhibition zone) and *Otiophora puciflora* (3 mm inhibition zone) as well as from *Celosia trigyna* L. against *Eurotium repens* in a 5 mm inhibition zone [50]. The most promising anti-plasmodial activity was found with extracts of *Cissampelos mucronata* with IC50 values of 1.8 µg/mL (EtOAC) and 1.1 µg/mL (MeoH) against a chloroquine-resistant strain (W2) and 2.9 µg/mL (EtoAc) and 1.5 µg/mL (MeOH) towards a chloroquine-sensitive strain (D6) [50]. The methanolic extracts of leaves and roots from three Nigerian medicinal plants including *Terminalia glaucescens* had antimicrobial activity against five clinical bacteria isolates comprising two Gram-positive bacteria (*Bacillus subtilis* and *Staphylococcus aureus*) and three Gram-negative bacteria (*Pseudomonas aerugenosis*, *E. coli*, and *Klebsiella pneumonia*) [32]. When acetone, methanol, and water extractions were investigated on *Peltophorum africanum*, *Ximenia caffra*, *Trichilia emetica* and *Terminalia sambesiaca*, the total yield in the acetone extract of *T. emetica* was high at 3.7% [42]. In the case of methanol extracts, *T. sambesiaca* had the highest yield (30.7%). The aqueous extract of *X. caffra* had the highest yield of 15.7%. Regarding the DPPH free radical scavenging activity of the extracts, the acetone and methanol extracts of *P. africanum* were more efficient in scavenging DPPH radicals, with IC50 of 13.00 ± 0.010 and 19.00 ± 010 µg/mL, respectively, while the aqueous extract of *T. emetica* had the best scavenging capacity (IC50 = 348.5 ± 0.077 µg/mL) compared to other solvents [42].

When Cameroonian medicinal plants were tested for correlating antioxidant compounds and anticancer properties, it was found that the crude methanol extracts from *Ekebergia senegalensis*, *Protea eliotii*, *Terminalia macroptera*, and *Vitellaria paradoxa* exhibited a high to moderate inhibitory effect on cell growth and were highly effective against the NCI-H460 cell lines, with GI50 values of 20.00 ± 0.04, 24.44 ± 0.90, 31.0 ± 1.5, and 27.0 ± 0.9 µg/mL, respectively [51]. Compared to other species, the highest antioxidant activity in the DPPH test was observed from the extracts of *Senna siamea*, *Eremomastax speciose*, and *Gardenia aqualla* [52]. In a study of antimalarial activity performed using Mali and Sao Tome medicinal plants, it was found that methanol extracts were effective only with *Ferteia apodanthera* leaves, which exhibited a pronounced antimalarial activity [51]. Previous studies on *Gardenia aqualla* revealed the presence of harman and tetrahydroharman, which are regarded as antioxidants because of their ability to scavenge free radicals and ability to inhibit lipid peroxidation. A study of South African medicinal grasses showed that methanolic root extracts of *Cymbopogon* spp., *Cymbopogon nardus*, and *Cenchrus cilliaris* had the highest total phenolic content, ranging from 4.2 to 30.9 mg GAE/g DW [53]. There was high DPPH free radical scavenging activity with an increase in extract concentration, whereby the EC50 values showed the best activity in *C. nardus* roots and inflorescences (0.02 and 0.04 mg/mL) compared to the other grass species. Out of 22 African tropical woody species from Ghana and Tanzania investigated for their biological activity, 13 species exhibited high antioxidant activity, with inhibitory DPPH radical scavenging concentrations (IC50) which were less than 10 µg/mL [54]. In that study, methanol crude extract from *Cylicondiscus gabunensis* was highly effective and exhibited antioxidant activity that was higher than that of (+)-catechin. The methanolic extraction of *Moringa oleifera* leaves was reported to contain chlorogenic acid, rutin, quercitin glucoside, and kaempferol rhanoglucoside, whereas in the root and the stem bark, only several procyanidin peaks were detected [55].

The extraction of plants using methanol is effective for certain plant species and various plant parts including leaves, stems, roots, or seeds [33]. This has been observed in different studies, but the success of extraction is species-dependent (Table 2). Notably, with regard to African medicinal plants further investigation is needed on the duration of methanolic extraction compared to other solvents.

## 5. Aqueous Extraction

Using water to extract chemicals from plants is a fundamental technique used in various fields like herbal medicine, food preparation, and botanical biochemistry [56]. This process is often referred to as water extraction or soaking, and there are various approaches depending on the type of plant material and the targeted chemicals to be extracted. The most reported approaches include infusion, decoction, cold water extraction, and steam distillation. When using water as a solvent, various factors determine the quality of extracts from plants, notably temperature, time, pH, and plant parts [57].

Pressurized hot water extraction has been reported as an effective method for extracting essential compounds such as water-soluble compounds like polyphenols, vitamins (especially vitamin C), and flavonoids [58]. Infusion refers to pouring water of different temperatures into a beaker containing the parts of the plant from which the antioxidant compounds are to be extracted. Various methods of infusion are the most common extraction methods for crude extracts. Infused hot water extraction was demonstrated to be effective in characterizing different medicinal plants for their phenolic compounds and potential response to cancer-related ailments [59]. The study was motivated by reports on various types of commonly used herbal teas that have been used for several decades. The high levels of polyphenols are dependent on the plant material’s preparation and the extract production techniques, aiming to yield optimum antioxidant compounds required to boost their potency [60]. Decoction methods are used to extract plant parts that are difficult to extract, such as roots, seeds, and bark [59]. The main elements of extraction from plants are minerals, bitter compounds, and some polysaccharides, and these are obtained in 15–60 min extraction time [61]. Decoction and boiling of plant parts to extract certain compounds are still widely used when formulating traditional herbal medicines that are useful in the treatment of various ailments such as anti-cancer [59,62]. This method provides the most abundant phytochemical levels in an eco-friendly and simplistic approach [62].

Maceration is an extraction method mainly for mucilage (from marshmallow root), enzymes, and some vitamins found in different types of medicinal and herbal plants. This method of extraction is relatively slow; however, if different solvents are added to the water in different combinations, better results are obtained. This was demonstrated with the extraction of *Silybum marianum*, whereby higher extraction yield was obtained from aqueous extracts (24.68%) followed by hydro-methanol (23.24%), hydro-acetonic (17.31%), and hydroethanolic (16.02%) solutions [63]. In the case of maceration, water was found to be the best extraction solvent with values of 24.71%, followed by methanol (22.03%), ethanol (19.92%), and finally, acetone. The same authors reported the highest polyphenols by maceration methods, whereby aqueous–methanol and aqueous–acetonic extracts had 18.75 ± 55 and 16.97 ± 5.46 mg GAE/g DW, respectively. The antioxidant activity of the aqueous–methanol and aqueous–ethanol extracts obtained by maceration was also high, with values above 73.84 GAE/g DW [63]. The sequential use of ethanol and aqueous maceration to extract phenolic compounds from *Ferulago angulata* resulted in high yield and antioxidant activity in lower ethanol dosages [64]. This finding demonstrates that the use of water extracts can replace the common use of alcohol and acid solvents, although extensive research is necessary. This is specifically important in African traditional medicine where water is more accessible to health practitioners.

Steam distillation is a process commonly employed in extracting volatile and aromatic compounds like essential oils, but it is limited when extracting vitamins and nutrient extracts without the use of inorganic solvents. This process is environmentally friendly and organic, and it ensures that the essential oils extracted are safe to use [65]. In studies conducted to extract essential oils from herbal and medicinal plants, 93% potential was reported with steam distillation whilst only 7% was found with other extracting methods [66]. Duration of the extraction process is key to the levels of compounds that are obtained. Biochemical components were demonstrated to increase rapidly during the first 4 h of extraction at 80 °C, and at a slower rate thereafter [66]. In contrast, at 20 °C, concentrations of all measured components were at or close to their maximum after 1 h and showed minor changes thereafter. Glucose and nitrate in the 20 °C extracts both declined substantially between 1 and 24 h [66]. Overall, that study demonstrated a positive effect on extraction using water at different temperatures to yield antioxidant compounds from various extracts. Commercially used extraction methods are expensive; traditional methods take longer but are still effective. Aquoeus extraction has been proven (Table 3). However, further studies are needed to explore aqueous extraction methods that are fast and obtain high yields of antioxidant compounds from different types of plants.

**Table 3:** Extraction of antioxidant compounds from African medicinal plants using water.

Plant Species	Extraction Method	Key Findings and Characteristics	Citation
*Moringa oleifera*; various herbal teas	Infusion (pressurized hot water extraction)	Effective for extracting water-soluble compounds like polyphenols, flavonoids, and vitamins. Considered organic and safe for consumption. Preparation techniques (temperature, time) are crucial for potency.	[58,60,67]
Various plants including *Adansonia digitata* L.; *Agrimonia eupatoria* Krylov; *Aloe ferox* (Mill.), *Aspalathus linearis* (Burm.f.) Dahlg.; *Eucomis autumnalis* (Mill.) Chitt.	Decoction	Used to extract minerals and bitter compounds. The process is simple and eco-friendly, providing accurate levels of compounds for treatments like anti-cancer applications.	[61,62]
*Catharanthus roseus*; *Ferulago angulata*	Cold water extraction (maceration)	A slow method used for delicate compounds like enzymes and some vitamins. Water can be used after maceration with other solvents like ethanol to improve results.	[64,68]
Aromatic and medicinal plants	Steam distillation	Extracts volatile and aromatic compounds (essential oils) without using organic solvents, ensuring safety. Achieved a high potential (93%) for extracting essential oils in some studies.	[65,69]

## 6. Application of Green Technologies in African Medicinal Plants and Challenges in Africa

There is active research into phytochemical extraction using “green” chemistry methods to address financial constraints and environmental concerns that result from traditional solvent-based extractions. Green extraction techniques focus on sustainability by reducing energy usage, reducing waste, and offering several advantages over traditional extraction techniques, such as reduced extraction time, lower solvent consumption, and higher yields of bioactive compounds [70,71]. Various innovative techniques have been developed, including ultrasound-assisted extraction (UAE) and microwave-assisted extraction (MAE) that break down plant cell walls and promote solvent penetration, which results in larger phytoextracts obtained in shorter periods [72,73,74,75]. The antioxidant activity of extracts obtained using MAE is generally high; for example, extracts from *Adansonia digitata* fruit pulp obtained using MAE exhibited significant antioxidant activity and epicatechin or (+)-catechin as the major compounds, particularly when using 50% ethanol as the solvent [76]. *Nymphaea lotus*, used traditionally in Africa, was subjected to UAE, which optimized extraction and significantly increased the flavonoid content and antioxidant potential compared to conventional methods [77]. In East Africa, UAE was used to extract methanolic compounds from the leaves and flowers of *Dodonaea angustifolia*, which exhibited significant antibacterial activity [78].

Traditional and green extraction techniques were used to extract bioactive compounds from *Cochlospermum planchonii*, an African medicinal plant commonly used to manage infectious diseases and inflammation [79]. The UAE yielded high total phenolic content and antioxidant activity, demonstrating its effectiveness in obtaining bioactive enriched fractions. Several studies indicate the potential of MAE and UAE for extracting bioactive antioxidant compounds (Table 4). However, the high temperatures and pressures during MAE can lead to the degradation of sensitive bioactive compounds, which is a significant concern with regard to maintaining the integrity of the extracted substances [78]. Similarly, the cavitation effect, which is central to UAE, can sometimes lead to the formation of free radicals and the degradation of thermo-sensitive compounds, affecting the quality of the extract [80,81]. Supercritical fluid extraction (SFE) is another notable green technique, which uses carbon dioxide to provide high selectivity and creates extracts without harmful waste as the solvent is easily eliminated through depressurization [71]. Moreover, technologies such as pressurized liquid extraction (PLE) and the use of natural deep eutectic solvents (NADESs) are gaining popularity. These technologies are known to produce high-quality extracts that are pure and have predictable chemical concentrations that make them ideal for usage in the food, cosmetic, and pharmaceutical industries.

The evident benefits from green extraction methods have been widely reported [84,85]. These have potential for the development of various products in food, environmental, pharmaceutical, and cosmetic industries [86]. Awad et al. [87] reported successful use of extracts from supercritical fluid extraction (SFE), pressurized liquid extraction (PLE), ultrasound-assisted extraction (UAE), microwave-assisted extraction (MAE), and pulse electric field-assisted extraction (PEFAE) as natural antioxidants in fresh meat. However, the literature on the development of secondary products from green extraction is very limited. The application of ‘green’ extraction technology in Africa is likely to be restricted by challenges like the high initial capital expenditure required for equipment such as SFE and PLE systems. This capital requirement can be restrictive for small to medium-sized businesses and many research institutes. Africa is also facing major infrastructural deficiencies in major service sectors, including the constant and stable supply of electricity in many areas. These deficiencies result in significant operational challenges for energy-dependent systems like MAE and UAE. The continent also faces a significant shortage of skilled labor and would require an additional force of competent staff to operate, maintain, and repair this complex technology. This would delay productivity from the time of purchase, deployment, and profitability. This delay highlights the need for increased localization of research innovations. A mismatch between academic research innovations and societies or locals impedes the transmission of new technologies to communities and enterprises that would profit most from the sustainable use of botanical resources.

## 7. Conclusions

The use of ethanol, methanol, water, and, to a less extent, acetone as solvents for extracting antioxidant compounds from African medicinal plants is popular. It has been indicated that different extraction solvents, concentrations, or techniques result in varying biochemical contents of extracts. It is evident from the findings that the selection of an appropriate extract assessment method is more important than choosing the plant part for the extraction. A direct proportional relationship has been noted between the concentration of the extract, the antioxidant compounds, and the antioxidant activity. Notably, more research needs to be conducted on the extraction duration and potential yield of antioxidant compounds from methanolic extracts, as well as antioxidant activity from ethanolic extracts. Fermented water extracts were reported to have higher antioxidant activity than fermented methanolic extracts from *Aspalanthus linearis* (rooibos). It is possible that alcohol molecules can aid in cellular penetration and cause cell-wall disruptions, leading to higher extract concentrations, but they contain chemicals that have oxidative effects over time. The use of ethanol after maceration in water to extract phenolic compounds and characterize antioxidant activity from *Ferulago angulata* (Chavir) resulted in improved outputs with a lower ethanol dosage. This finding shows that water can replace typical extraction with high dosages of alcohol solvents if plant samples are pre-processed for high solubility, although extensive research is necessary. This is particularly essential on continents such as Africa, where water is abundant, but alcohol is scarce. Further research is required to investigate the development of other quick aqueous extraction technologies capable of extracting high-quality extracts with considerable amounts of phytochemicals for use with African herbs. Future studies should include statistical correlations of extract properties and their antimicrobial activities. This can guide farmers of medicinal plants in using the antioxidant compounds’ content to target a specific phytomedicinal industry.

## Figures and Tables

**Table 2:** Extracted antioxidant compounds of African medicinal plants by methanolic solvents.

Plant Species	Solvent	Key Findings	Citation
*Amaranthus hybridus*	Methanol	The leaf extract (40.1 mg GAE/g DM) had a higher total phenolic content than the seed extract (31.20 mg GAE/g DM).	[33]
*Anacardium occidentale*	Methanol	The stem bark extract showed a high total phenolic content (660.52 mg GAE/g DM).	[33]
*Calpurnia aurea*	Methanol	Showed substantial phenolic compounds and were fast and effective scavengers of ABTS radicals, comparable to BHT.	[34]
*Leonotis leonorus*, *Solanum nigrum*	Acetone, methanol, and water	Methanol extracts had higher proanthocyanidin content.	[35]
*Tetrapleura tetraptera*, *Parkia biglobosa*	Methanol, ethanolic, and water	Methanolic extracts exhibited higher antioxidant capacity, which correlated with higher polyphenol content.	[36]
*Psorospermum febrifugum*, *Myrianthus arboreus*	Methanol, dichloromethane	Methanolic extraction of *P. febrifugum* yielded the highest amount (30.8%) and showed strong antioxidant activity, better than ascorbic acid.	[36]
*Aspalathus linearis* (Rooibos)	Methanol and water (fermented vs. unfermented)	Unfermented methanol extracts produced higher total phenolic content than fermented extracts.	[37]
*Bergenia ciliata*	Methanol	Showed the strongest antioxidant activity among the three plants tested, with an IC_50_ value close to L-ascorbic acid.	[38]
*Mesembryanthemum edule* L.	Methanol and Ethanol	Methanolic extraction was richer in total polyphenols. Increased sonication time enhanced polyphenol content.	[39]
*Napoleona imperialis*	Methanol	Inhibited the growth of 11 bacterial strains and demonstrated wound healing properties.	[43]
*Cymbopogon nardus*	Methanol	Had the highest total phenolic content and showed the best free radical scavenging activity amongst the investigated grasses.	[53]
*Cylicondiscus gabunensis*	Methanol	Showed the highest antioxidant activity, even higher than the standard catechin.	[54]

**Table 4:** Application of Ultrasound-Assisted Extraction (UAE) and Microwave-Assisted Extraction (MAE) for extracting antioxidant compounds from African medicinal plants.

Plant Species	Extraction Method	Compounds Extracted	Properties	Reference
*Adansonia digitata*	MAE	Polyphenols, flavonoids	High polyphenolic content; improved antioxidant activity	[76]
*Nymphaea lotus*	UAE	Flavonoids	Increased flavonoid content; enhanced antioxidant potential	[77]
*Dodonaea angustifolia*	UAE	Phenolic acids, flavonoids	Significant antibacterial activity	[78]
*Plectranthus madagascariensis*	MAE	Caffeic acid, chlorogenic acid, rosmarinic acid	Potent antibacterial activity; significant antioxidant activity	[82]
*Cochlospermum planchonii*	UAE	Phenolics, flavonoids	High phenolic content; strong antioxidant activity	[79]
*Gloriosa superba*	MAE	Colchicine	High colchicine yield	[83]

## Data Availability

No new data were created or analyzed in this study. Data sharing is not applicable to this article.
